# Convolutional and recurrent neural network for human activity recognition: Application on American sign language

**DOI:** 10.1371/journal.pone.0228869

**Published:** 2020-02-19

**Authors:** Vincent Hernandez, Tomoya Suzuki, Gentiane Venture

**Affiliations:** GVLAB - University of Agriculture and Technology of Tokyo, Tokyo, Japan; Newcastle University, UNITED KINGDOM

## Abstract

Human activity recognition is an important and difficult topic to study because of the important variability between tasks repeated several times by a subject and between subjects. This work is motivated by providing time-series signal classification and a robust validation and test approaches. This study proposes to classify 60 signs from the American Sign Language based on data provided by the LeapMotion sensor by using different conventional machine learning and deep learning models including a model called DeepConvLSTM that integrates convolutional and recurrent layers with Long-Short Term Memory cells. A kinematic model of the right and left forearm/hand/fingers/thumb is proposed as well as the use of a simple data augmentation technique to improve the generalization of neural networks. DeepConvLSTM and convolutional neural network demonstrated the highest accuracy compared to other models with 91.1 (3.8) and 89.3 (4.0) % respectively compared to the recurrent neural network or multi-layer perceptron. Integrating convolutional layers in a deep learning model seems to be an appropriate solution for sign language recognition with depth sensors data.

## Introduction

Sign language is a language that mainly uses hand kinematics and facial expressions. It is widely used by hearing-impaired people to communicate with each other, but rarely with people who do not have hearing impairment. Therefore, they are only in direct contact with hearing-impaired persons, which greatly limits social interactions. An alternative would be to have a real-time translation with interpreters, but they are not permanently available and can be rather expensive. Therefore, a system that could enable automatic translation would be of great interest.

Human Activity Recognition (HAR) in general is an important and challenging topic o address because of the large variability that exists for a given task. Indeed, whether the variability comes from a subject repeating an action several times or more importantly between subjects, the kinematics behavior over time presents a certain challenge to generalize. HAR considers that these behaviors are represented by specific patterns that could be classified using machine learning algorithms.

Nowadays, human movement data can be easily extracted from low-cost systems that integrate depth mapping sensors such as Kinect and LeapMotion. These systems are ready to use, require a relatively short set-up time and the data can be easily extracted. Therefore, they can be easily used to quickly acquire a large amount of data, which is a requirement when considering machine learning. Other approaches were considered with sEMG [1], CyberGloves [2] or motion capture system but these techniques are difficult to use outside of laboratories.

Considering systems such as the Kinect® or a combined Kinect/LeapMotion® approach, several studies have been conducted using video or depth-mapping sensor and machine learning approaches such as Hidden-Markov-Model (HMM) [3], coupled HMM [4], random-forest per-pixel [5], multi-class Support Vector Machine (SVM) [6], linear binary SVM [7], convolutional neural network (ConvNet) [8,9] and DeepConvLSTM video-based approaches [10] to name a few. Such approaches are interesting, but the Kinect remains difficult to use in public space since it requires a large space and a power supply.

The LeapMotion easily detects forearm and palm movements as well as the position of the fingers and thumb with a measurement accuracy of 200 μm [11] to estimate the joint position. Such accuracy can be useful for creating accurate models of the kinematics of the hand-arm system. In addition, it can be used with a single USB port, consumes very little power, and does not require a large space to be used, making it convenient for applications in off-lab environments. Moreover, it is an affordable system that does not require a professional to set up and no calibration is required, which makes the system practical to use. In addition, the development of depth-sensing cameras on smartphones would be an interesting choice as a basis for sign language recognition and for the development of portable systems that are easy to use.

Naidu and Ghotkar [12] used the LeapMotion to classify a subset of the Indian Sign Language (10 Arabic numbers, 26 letters, and 9 words). They propose four different approaches (Euclidian distance measure, similarity, Jaccard and dice similarity) with 8 distances computed from 6 measured points (center of the palm and all fingertips). Cosine similarity showed an accuracy of 90.00% for the complete dataset but their approach is limited to static posture. Marin et al. [6] studied a combined LeapMotion/Kinect approach with multi-class SVM to classify 10 static American Sign Language (ASL) words with manual extraction of key moments. They presented an average accuracy of 80.86%, 89.71% and 91.28% with the LeapMotion, the Kinect and combined Kinect/LeapMotion approaches respectively with a user-independent *k*-fold cross-validation (M-1 subject for each K) for parameters tuning. Then, the classifier was trained again with all subjects (M) and computed the accuracy on it which does not provide a reliable model that can be used with new subjects. Kumar et al. [4] also considered a combined Kinect/LeapMotion approach on 25 dynamics words of the Indian Sign Language. They considered a coupled-HMM approach and gained 90.8% accuracy on 25% out of the complete dataset (50% used for training and 25% for parameter tuning and parameter validation), however, they do not provide information whether or not a user-independent test was considered. Chuan et al. [13] classified the 26 letters of the English alphabet using k-NN and SVM classifiers. Results showed an average classification rate of 72.78% and 79.83% respectively using *k*-fold cross-validation (K = 4) on the complete dataset composed of 2 subjects. Fok et al. [14] proposed an HMM-based approach to recognize the 10 ASL Arabic numbers with an overall average recognition of 93.14% half of the sample of each subject for training and the remaining data to test the model.

The recent breakthrough of deep learning has outperformed conventional machine learning approach in computer vision tasks [15]. Deep learning uses a succession of layers to extract information, with the output of one layer as the input of the following one. They provide a robust approach to generalize inter-subject variability and can consider the time-series dynamics behavior of human movements mainly with ConvNet and Recurrent Neural Network (RNN). Regarding sign language recognition, Koller et al. [16] use a hybrid ConvNet-HMM approach based on a sequence of images extracted from video and by tracking the dominant hand.

Regarding deep learning, the state of the art on HAR is presented by Ordóñez and Roggen [17] adapted from Sainath et al. [18] for speech recognition. They proposed a model based on combining convolutional and recurrent layers with Long-Short-Term-Memory (LSTM) called DeepConvLSTM with data from Inertial Measurement Unit sensors (IMUs). ConvNet and RNN have supervised learning approaches that can learn the dependencies between given inputs and outputs. DeepConvLSTM outperformed ConvNet and RNN considered independently for speech recognition [18] and is considered as the state-of-the-art for HAR [17,19]. Indeed, both approaches have their advantages with convolutional layers that can extract features from a given signal and recurrent layers that can consider the dynamics of a time-series signal [20–22]. A combined approach seems to be the best approach to perform HAR and is promising since it allows flexible data fusion and features extraction [17] and this approach will be exploited in this study.

This research proposes a comparison of deep neural networks to classify 60 ASL signs based on a complete kinematic model of the right and left forearm/hands/fingers/thumb. The kinematics of the hand and forearm are derived from the skeletal tracking model provided by the LeapMotion sensor. The purpose of this research is also to propose a robust user-independent *k*-fold cross-validation and tests in contrast to previous studies that focused primarily on intra-user testing of their models. Indeed, machine learning models can be representative of the behavior of participants but not on new ones. It is also demonstrated that intra-user tests can lead to an overestimation of accuracy that is not representative of the results that can be obtained on new users.

This document is organized as follows. We present the experiment as well as details on the extraction and processing of the data prior to their use in our models. Then, we present the classification methods of the gestures, the learning properties of the models and the hyperparameters used. Finally, the results comparing the different models are presented and discussed.

## Material and methods

### Experimentation

The experiment was approved by the local ethics committee at the Tokyo University of Agriculture and Technology (TUAT) in Koganei, Japan. All Experiments were done in 2018. Before the experiment, the participants gave informed consent to participate in the study.

A dataset of 25 male subjects, all novices in any sign language, was collected. Before each measurement, the corresponding sign was taught to them. In this study, the Arabic numbers from 0 to 10 and 49 words were considered. The total numbers of each sign gathered are presented in Table 1.

**Table 1:** Numbers of sign gathered during the experiment with the 25 subjects.

Dataset (25 subjects)											
Sign	#	Sign	#	Sign	#	Sign	#	Sign	#	Sign	#
**0**	285	**Big**	251	**Come**	296	**Green**	268	**Red**	272	**Where**	256
**1**	322	**Blue**	239	**Cost**	290	**Happy**	275	**Shoes**	311	**Why**	254
**2**	308	**Brush**	275	**Cry**	280	**Hot**	288	**Small**	279	**With**	305
**3**	277	**Bug**	252	**Dad**	278	**Hungry**	291	**Socks**	265	**Work**	289
**4**	296	**Candy**	258	**Deaf**	223	**Hurt**	286	**Stop**	277	**Yellow**	247
**5**	320	**CarDrive**	250	**Dog**	272	**Milk**	301	**Store**	272		
**6**	299	**Cat**	262	**Drink**	280	**Mom**	298	**Thanks**	270		
**7**	283	**Cereal**	250	**Egg**	307	**More**	315	**Warm**	299		
**8**	275	**Clothes**	292	**Finish**	323	**Orange**	287	**Water**	260		
**9**	297	**Coat**	283	**Go**	293	**Pig**	277	**What**	287		
**10**	260	**Cold**	345	**Good**	315	**Please**	262	**When**	263	**Total**	16890

### Features extraction

The LeapMotion SDK Unity core assets 4.3.2 was used with the C#/Unity module to collect data during the experimental protocol. A total number of 26 points on each hand are extracted in real-time from the LeapMotion. The features computed from these points are based on a multi-finger modeling approach presented by Carpinella et al. [23] with minor modifications and the ISB recommendations for the relative orientation of the hand to the forearm [24].

### Kinematics models

**Fig 1:**
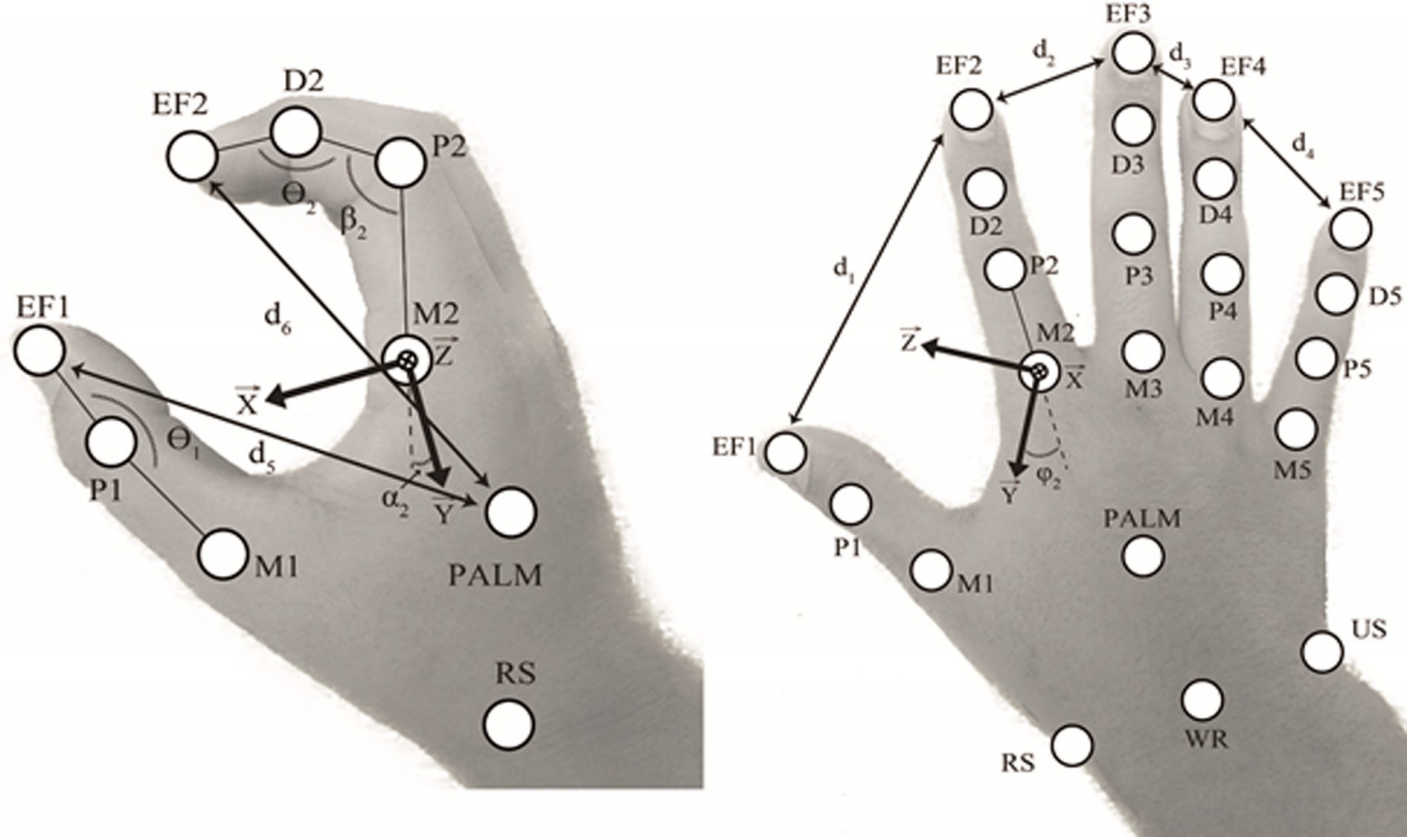
Hand joints. Thumb, index, middle, ring, and pinky are numbered from 1 to 5 respectively. EF_*i*_ (*i* = 1–5): finger and thumb end-effector position, M_*i*_ (*i* = 1–5): finger and thumb metacarpophalangeal joint, P_*i*_ (*i* = 1–5): finger and thumb proximal interphalangeal joints, D_*i*_ (*i* = 2–5): finger distal interphalangeal joints. EL: elbow joint (not represented here), WR: wrist joint, RS: radial styloid, US: ulnar styloid, PALM: palm center.

The following coordinates systems are considered for the hand (1) and forearm (2) (Fig 1). (1) $\vec{Z}$ has the direction of (M_*2*_-M_*5*_) pointing externally, $\vec{X}$ is perpendicular to the plan form by (M_*2*_-RS) and (P_*5*_-RS) pointing forwardly, and $\vec{Y}$ is the cross product of $\vec{Z}\times \vec{X}$ pointing upwardly. (2) $\vec{Y}$ has the direction of (EL-US) pointing upwardly, $\vec{X}$ is the cross product of $\vec{Y}$ and (RS-US) pointing forwardly and $\vec{Z}$ is the cross product of $\vec{X}$ and $\vec{Y}$ pointing externally. Then, the Euler angles that describe the relative orientation of the hand with the forearm (flexion/extension and radial/ulnar deviation) are computed with a **ZXY** rotation sequence (Vectors direction are expressed from the standard anatomical position).

The following kinematics are considered for the thumb and fingers (thumb: *i* = 1, index: *i* = 2, middle: *i* = 3, ring: *i* = 4 and pinky: *i* = 5) with EF_*i*_ (*i* = 1–5) that represents the finger and thumb end-effector, M_*i*_ (*i* = 1–5) the finger and thumb metacarpophalangeal joint, P_*i*_ (*i* = 1–5): the finger and thumb proximal interphalangeal joints and D_*i*_ (*i* = 2–5) the finger distal interphalangeal joints. Then, the relative orientation of each finger with the hand is represented with a flexion angle α_i_ (Angle between $\vec{Y}$ and M_*i*_ - P_i_ projected in the **XY** plan) and an abduction angle φ_i_ (Angle between $\vec{Y}$ and M_*i*_ - P_i_ projected in the **YZ** plane). Moreover, the relative orientation of the thumb with the hand is represented with a flexion angle α_1_ (Angle between $\vec{Y}$ and M_*i*_ - D_i_ projected in the **YZ** plan) and an abduction angle φ_1_ (Angle between $\vec{Y}$ and M_*i*_ - D_i_ projected in the **XY** plane). Furthermore, the angle β_i_ between P_*i*_ - D_*i*_ and P_*i*_ - M_i_ and the angle ϴ_i_ between EF_i_ - D_*i*_ and P_i_ - D_*i*_ for the *i*^*th*^ finger (*i* = 2–5) and the angle ϴ_1_ between EF_1_ - D_1_ and M_1_ - D_1_ for the thumb are also computed. Finally, d_*j*_ (*j* = 1–9) represent respectively the Euclidian distance between each successive EF (EF_1_ with EF_2_, EF_2_ with EF_3_ …) and between EF_*i*_ (*i* = 1–5) and the Palm.

### Dataset

Each sign is manually extracted and interpolated to 100 frames with cubic spline interpolation. The dataset is composed of a total number of *d* = 16890 labeled signs. *f* = 60 features are computed to represent the kinematics of the right and left sides (Fig 1). For each sign extracted, the features were regrouped in a time-series matrix **F**_*l*_ $\in {\mathbb{R}}^{m\times n}$ (*m* = 100 and *n* = 60) with each group given by: (1) the finger and thumb end-effector (EF) relative distance, (2) their distance with the palm, (3) the relative orientation of the hand with the forearm, (4) the relative orientation of the fingers and thumb with the hand and (5) the thumb and finger angles.

$$F_{l}=\left[\begin{array}{cccc} f_{1,1} & f_{1,2} & \ldots & f_{1,i}\\ f_{2,1} & f_{2,2} & \ldots & f_{2,i}\\ \ldots & \ldots & \ddots & \ldots \\ f_{t,1} & f_{t,2} & \ldots & f_{t,i} \end{array}\right]=f_{i,j}\in {\mathbb{R}}^{m\times n}$$ (1)

All feature matrices were computed for all signs (*l*) and subjects (*k*). Finally, each feature *f*_*i*,*j*_ was standardized with the mean *μ* and standard *σ* deviation of their respective group presented above as follow:

$$f_{i,j}=\frac{f_{i,j}-\mu }{\sigma }$$ (2)

Then, each subject in the dataset is represented as ${\text{D}}_{s}={\left\{F_{l},y_{l}\right\}}_{l=1}^{N}$ with *N* the numbers of sign and **y**_*l*_ the corresponding outputs labels of **F**_*l*_ represented as a binary vector.

### Data augmentation

To prevent the overfitting of the model and help generalize the classification, data augmentation was considered. A given signal **f**_*i*_ is sliced into 10 parts of equal length. Then, data warping was considered on the magnitude and the temporal location of the signal with the following properties. First, each part was distorted to a random value *p* {p∈ℕ|5≤i≤15} with a cubic spline interpolation and all parts were reconstructed and interpolated to 100 frames. Then, the amplitude of the signal was slightly altered. A sinus wave with a random period *P* {P∈ℝ|0.5≤P≤2}, phase φ {φ∈ℝ|0≤φ≤π} and amplitude *A* {A∈ℝ|−0.2≤A≤0.2} was generated. Finally, this sinus wave was multiplied by *A* and by the amplitude range of the current signal **f**_*i*_ and added to it, thus allowing a smooth variation. This procedure is repeated 40 times for each **F**_*l*_ in the training set (described in the next section).

## Gesture classification

### Training, validation, and test

This study considers a user-independent approach, i.e. data from the same participant appears either in the train, validation or test sets. To do so, nested *k*-fold cross-validation with a user-independent approach is considered. It consists of an inner and an outer loop. In each loop, the model is always trained from a random initial point to keep each inner and outer loop independent from each other. Moreover, the first outer loop is considered for hyper-parameters tuning (e.g. number of layers, cells unit size, learning rate, strides, patch size…).

The inner loop consists of splitting the participants into two sets: 20 subjects are assigned to the training/validation set and 5 to the test set. The test set was kept aside to assess the final performance of the models. The training/validation set is used in a non-exhaustive user-independent *k*-Fold cross-validation that consisted of a rotating K = 4 folds with 15 and 5 subjects to train and validate respectively. To reduce overfitting of the neural network, early-stopping is applied at each *k*-fold when the accuracy of the validation set starts to decrease (overfitting of the model on the training set).

The outer loop follows the same procedure as the inner loop except that the test set is changed (as well as the train/validation one) with user that still appeared once either in the train/validation or test set. This loop is performed until all participants are tested.

### Training properties

Models were trained with mini-batches composed of 500 samples and a learning rate of η = 0.001 with exponential decay of 0.9 every 5 epochs. As a form of regularization, a dropout wrapper is added on each layer to randomly select units that are ignored at each epoch with a probability value of 0.8. The Adam gradient descent optimization algorithm [25] was used to minimize the cost function *E* that corresponds to a softmax cross-entropy between the estimated vector (logits) (**y’**) and the true labels vector (**y**):

$$\begin{array}{l} E=-\sum_{i=0}^{n}y_{i}^{'}\text{log}\left(\text{softmax}(y_{i})\right)\\ \text{with softmax}(y_{i})=\frac{\text{exp}(y_{i})}{\sum_{i=0}^{n}\text{exp}(y_{i})} \end{array}$$ (3)

For each *k*-fold cross-validation, the training phase was stopped when the accuracy of the validation set starts to decrease and the corresponding model was saved.

After the *k*-fold cross-validation in each outer loop, 4 trained models are created. The test set is fed to each model (*k* = 4) and the final predicted class is considered with a majority vote decision.

### Models

**Fig 2:**
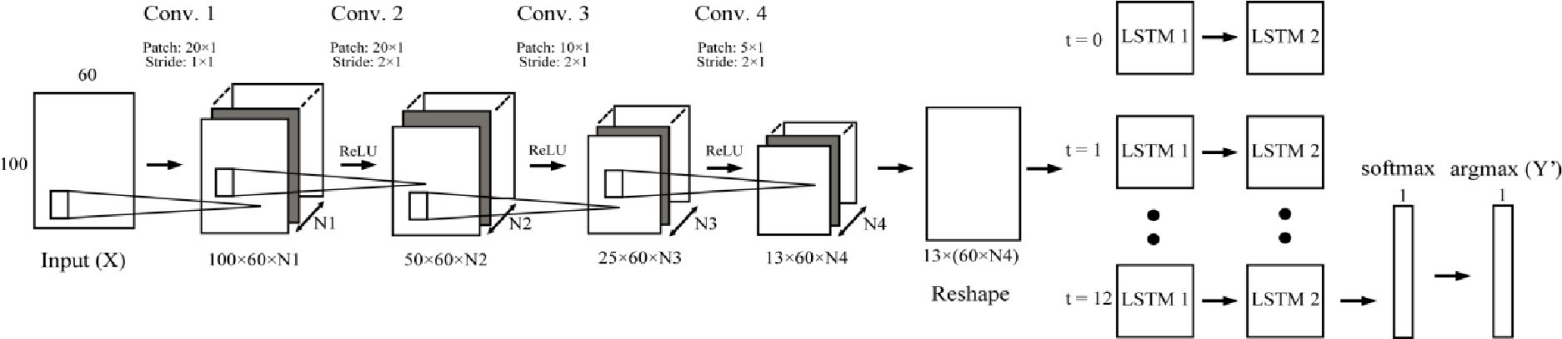
DeepConvLSTM architecture. ConvNet-LSTM architecture. N1, N2, N3, and N4 represent the numbers of channels (also called feature map) created after each convolutional layer. Their values are respectively 16, 16, 32 and 64. ReLU (rectified linear unit) represent the activation function.

In this study, different conventional machine learning and deep learning models are considered. Regarding the conventional machine learning models, k-nearest neighbors (*k*-NN) [26], random forest (RF) [27] and support vector machine (SVM) [28] are used as a baseline comparison. The retained hyperparameters are as follows: *k*-NN is used with *k* = 10, RF is used with 1000 trees classifier with a depth of 5 and SVM is used with a linear kernel. The deep learning models considered were Multi-layer Perceptron (MLP), ConvNet, RNN with LSTM cell from Zaremba et al. [29] and DeepConvLSTM (Fig 2). Detailed regarding their hyperparameters is provided in Table 2.

**Table 2:** Detail of the hyperparameters retained for Multi-layer Perceptron (MLP), convolutional neural network (ConvNet), recurrent neural network (RNN) and DeepConvLSTM.

	MLP	ConvNet	RNN	DeepConvLSTM
Input shape	6000	100 x 60 x 1	100 x 60	100 x 60 x 1
Layers 1	Neurons: 256 Activation function: Sigmoid	Filter size: 16 Kernal size: 20 x 1 Stride: 1 x 1 Activation function: ReLU	Cells: LSTM Cells size: 256	Filter size: 16 Kernal size: 20 x 1 Stride: 1 x 1 Activation function: ReLU
Layers 2	Neurons: 512 Activation function: Sigmoid	Filter size: 16 Kernal size: 20 x 1 Stride: 2 x 1 Activation function: ReLU	Cells: LSTM Cells size: 256	Filter size: 16 Kernal size: 20 x 1 Stride: 2 x 1 Activation function: ReLU
Layers 3	Neurons: 1024 Activation function: Sigmoid	Filter size: 32 Kernal size: 10 x 1 Stride: 2 x 1 Activation function: ReLU	Cells: LSTM Cells size: 256	Filter size: 32 Kernal size: 10 x 1 Stride: 2 x 1 Activation function: ReLU
Layers 4	Neurons: 60 Activation function: Softmax	Filter size: 64 Kernal size: 5 x 1 Stride: 2 x 1 Activation function: ReLU	Cells: LSTM Cells size: 256	Filter size: 64 Kernal size: 5 x 1 Stride: 2 x 1 Activation function: ReLU
Layers 5		Neurons: 128 Activation function: tanh	Neurons: 60 Activation function: Softmax	Cells: LSTM Cells size: 256
Layers 6		Neurons: 60 Activation function: Softmax		Cells: LSTM Cells size: 256
Layers 7				Neurons: 60 Activation function: Softmax

Tensorflow 1.4 [30] with Python 3.5.2 was used and the training was performed on a GTX 1060 6GB that integrates 1280 CUDA cores [31].

Specifically to DeepConvLSTM, the output of the last convolutional layers is fed into 2 LSTM layers composed of the LSTM cell from Zaremba et al. [29]. Moreover, as the time-series is fed to the LSTM layers, the LSTM hidden state contains more and more information about the current sequence. Therefore, the last time step of the LSTM layer is connected to the softmax layer to retrieve the class probabilities. Finally, the last output of the last recurrent layers is connected to a layer with a softmax activation function to normalize the output to a probability distribution.

## Results

The results from all different models considered with and without data augmentation are presented in Table 3. To compare the results of the different approaches, a one-way Analysis of Variance (ANOVA) with repeated measures was performed. Then, the Dunnett post hoc test was used to compare the DeepConvLSTM with data augmentation with all other models. The significance level was set at α = 0.05 and the Statistica software (Statsoft, Tulsa, OK, USA) was used. ANOVA on models showed a significant main effect (F(10, 264) = 42.454, p < 0.05) and Dunnett’s test revealed higher value of accuracy for DeepConvLSTM with data augmentation compared to all other models (p < 0.05 for each comparison) except with ConvNet with data augmentation (p > 0.05).

**Table 3:** Accuracy (Mean (SD)) on the validation and test set for the different models considered (+DA: Include data augmentation). The results include all inner and outer loops of the nested *k*-fold cross-validation (* p < 0.05).

	*k*-fold cross-validation	Test
DeepConvLSTM (+DA)	89.7 (2.1)	**91.1 (3.8)**
LSTM (+DA)	83.0 (3.5)	87.2 (5.1) *
ConvNet (+DA)	86.7 (2.7)	89.3 (4.0)
MLP (+DA)	81.3 (3.4)	84.5 (5.0) *
DeepConvLSTM	85.3 (1.9)	87.3 (4.5) *
LSTM	79.4 (2.7)	84.5 (5.4) *
ConvNet	83.7 (1.9)	87.4 (4.3) *
MLP	80.8 (2.4)	83.7 (5.0) *
k-NN	65.4 (2.9)	67.1 (6.4) *
RF	78.1 (2.5)	80.1 (4.9) *
SVM	77.7 (3.1)	80.6 (4.9) *

The normalized confusion matrix and a Sankey diagram created using SankeyMATIC (http://sankeymatic.com/) are presented in the supplementary materials S1 and S2 Figs in the supplementary materials respectively.

Moreover, the *holdout method* with the same DeepConvLSTM hyperparameters without data augmentation was also considered. It simply consists in splitting the complete dataset into two parts randomly selected (80% training and 20% testing) in a stratified way (the same percentage of labels in each set). The *holdout method* was repeated 10 times and results showed a mean accuracy of 0.997 (0.001) with the test set.

## Discussion

The aim of this study was to classify 60 signs of ASL using deep neural networks with a forearm, hand, and finger kinematics models from joint position data provided by the LeapMotion. The raw data from the LeapMotion for the 25 subjects are freely provided with this study (https://doi.org/10.17632/8yyp7gbg6z.1). Moreover, a robust user-independent *k*-fold cross-validation was used to create the model. This step was composed of four cycles (*k* = 4) with 15 subjects for training and 5 subjects for validation without overlap. It was followed by a user-independent test phase with 5 subjects used to apply the model in a “real-world case” to present the classification results. Moreover, the DeepConvLSTM was compared with a recurrent neural network composed of the LSTM cell from Zaremba et al. [29] to demonstrate the improvement of a combined approach.

Results showed that the DeepConvLSTM with data augmentation showed the highest results with 91.1 (3.8) % of accuracy on the test sets. The effect of data augmentation on this model showed a significant improvement of about 3.8% (Table 2) demonstrating the importance of using data augmentation technique to help the classifier to generalize human behavior [32]. ConvNet with data augmentation showed non-significant different results compare to DeepConvLSTM with an accuracy of 89.3 (4.0) % and RNN presented a lower accuracy of 87.2 (5.1) % (Table 2). Contrasting with the study of Ordóñez and Roggen [17], the DeepConvLSTM did not outperform ConvNet but outperformed RNN demonstrating the importance of using convolutional layers for HAR. MLP, k-NN, SVM, and RF showed poorer results compared to models composed of convolution or recurrent layers. The current approaches considered the use of kernel size in the convolutional layers along the time axis (e.g. 20 x 1 on the first convolutional layer). Using a larger kernel or changing the input tensor size to 100 x 1 x 60 to get the first convolution to capture all the vector interaction was also tested but was not as successful. It may be difficult for the convolutional layer to capture 60 dimensions along the feature axis at once. Convolutional layers are used to remove outliers’ values, extract features and filter the input data independently along the time axis, making the classification more efficient in the classifier part of the models. RNN models with LSTM cells reach high accuracy (87.2 (5.1) % with data augmentation) which are improved by the convolutional layer in the DeepConvLSTM models. Nevertheless, using a 1D kernel on each feature independently along the time axis in this study provided the best results. As also pointed out by Ordóñez and Roggen [17], using max-pooling at each convolutional layers provided poorer results. Moreover, the test time was about 15.3 (0.6)s and 8.8 (0.3)s. for 5 tested subjects in all outer loop for the DeepConvLSTM and ConvNet respectively providing an advantage of using ConvNet for portable systems.

Regarding the *holdout method* (used for demonstration purpose only), the accuracy on the validation set was at 99.7 (0.1) %. This result showed how easy it is to gain very high accuracy. This method should be avoided since the models have already learned all the subject’s behavior and the results would remain specific to these subjects and would not be representative of new users. Moreover, other DeepConvLSTM hyperparameters that provided lower accuracy with the user-independent approach also provided accuracy around 99% with the *holdout method* demonstrating the risk to create models with non-adapted hyperparameters. It remains difficult to compare our results with those of previously reported studies since they used different validation and/or test methods, but we believe that considering a user-independent approach with nested *k*-fold cross-validation is a relevant way to prove the reliability of a model.

The Sankey diagram (S2 Fig) shows for each true label **y** the wrong logits **y’** that has been identified in an easy-to-read manner. For a better reading, the wrong logits mislabelled 1 and 2 times for each label and subject independently were removed from the Sankey Diagram. Some movements of the sign language were confused because of similarities between them or difficulty from the LeapMotion to correctly detect all joint positions. For instance, *Two* was confused with *Three*, *Seven* with *height* and *Four* with *Five*. Indeed, it was sometimes difficult for the LeapMotion sensor to differentiate the numbers of extended fingers. Movement such as *dog* (forearm in supination with index finger extended and snap the thumb with the middle finger) is difficult to record by the LeapMotion since the thumb and the middle finger are hidden by the palm and can be confused with one (index finger extended) and brush (index finger extended moving in front in the mouth). *Thanks* consist of only moving the forearms with the hand flat and finger close to each other while *warm* consists of the same movement with finger performing and abduction during the movement. The model may receive too limited information to perfectly differentiate both signs. The same problem is observed with *car*, *coat*, and *cold* since they are movements that require moving the forearms with the fists closed which may also provide too limited information given the model here used to be differentiated properly. Finally, *Dad* was confused with *Mom* since they required the same hand movement to be performed except that the position in front of the head is different from the thumb position at the chin and at the forehead respectively. Despite this, *Dad* was confused with *Mom* for 23% in the test set showing the capability of the DeepConvLSTM to differentiate the slight difference in the global dynamics of the movement. Improvement in the hand tracking with depth-sensor may leverage these problems and would help to reach higher accuracy in the future.

The main limitation of this study is that the recruited participants were new to sign language. Beginners may have greater variability when performing movements and future work should be considered with experts to assess if there would be an improvement in the classification. In addition, a static neural network was used and future work will have to be considered with a dynamic neural network that allows adapting the length of the input sequence coupled with an automatic signal detection method [33]. Nevertheless, a static neural network may still be considered with an automatic segmentation method and then interpolate the signal. Finally, future studies should consider another model that will be fed by the output of the DeepConvLSTM with the purpose of predicting the most probable word based on the previous words/sentences to help the classification from a given context. Finally, sign language recognition may be improved with data provided by a camera or IMU sensors to gain information about the hand position relative to the body.

## Conclusion

This study compared various conventional machine learning and deep learning models to classify American sign language. Moreover, a robust user-independent *k*-fold cross-validation and test phase were provided. This contrast previous work, where the validation and/or the test phase were not user-independent, or lack of information was provided. There are several possibilities for future work to improve these results, such as the use of experts in sign language, dynamic neural network, automatic segmentation technique and additional data from a camera or IMU sensors.

## Supporting information

S1 Fig Normalized confusion matrix. (DOCX)

S2 Fig Sankey diagram. (DOCX)
