# Supplementary material for: Convolutional and recurrent neural network for human activity recognition: Application on American sign language
Source: PLoS One. 2020 Feb 19;15(2):e0228869. doi: 10.1371/journal.pone.0228869 (PMC7029868; doi:10.1371/journal.pone.0228869)
Supplement: S1 Fig — (DOCX) [file pone.0228869.s001.docx]

**Appendix A. Normalized confusion matrix**

**

**
