# Supplementary figures and images for: Convolutional and recurrent neural network for human activity recognition: Application on American sign language
Source: PLoS One. 2020 Feb 19;15(2):e0228869. doi: 10.1371/journal.pone.0228869 (PMC7029868; doi:10.1371/journal.pone.0228869)

**Appendix B. Sankey diagram**

**
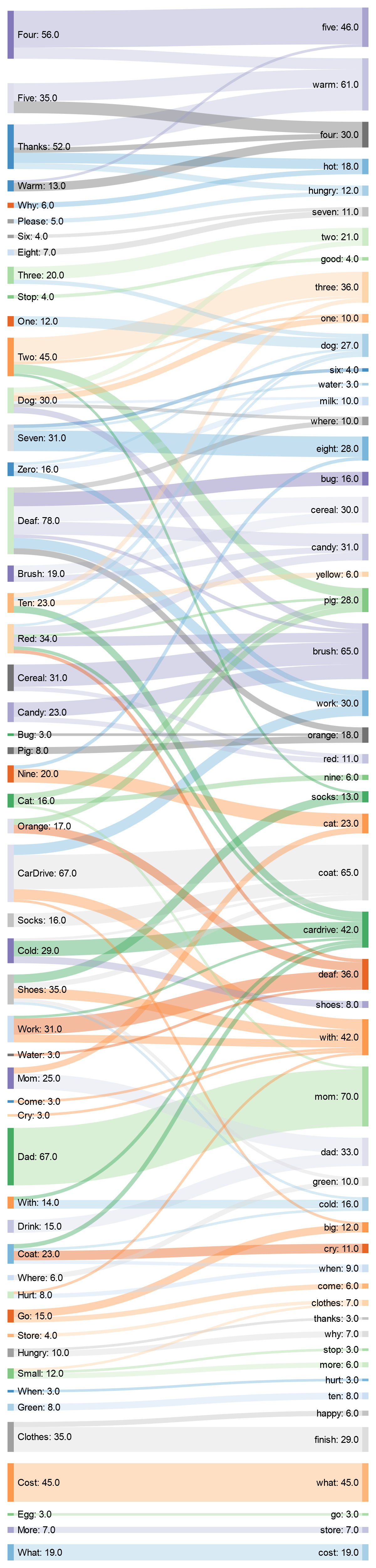
**

Supplement: S2 Fig — (DOCX) [file pone.0228869.s002.docx]
